# Co-occurrence of thelytokous and bisexual *Trichogramma dendrolimi* Matsumura (Hymenoptera: Trichogrammatidae) in a natural population

**DOI:** 10.1038/s41598-019-53992-8

**Published:** 2019-11-25

**Authors:** Quan-quan Liu, Jin-cheng Zhou, Chen Zhang, Su-fang Ning, Li-jia Duan, Hui Dong

**Affiliations:** College of Plant Protection, Shenyang Agricultural University, Shenyang, Liaoning China

**Keywords:** Microbiology, Physiology

## Abstract

*Trichogramma dendrolimi* is one of the most successful biocontrol agents in China. However, an inundative condition is necessary to obtain acceptable parasitism effect. A good solution to this is the application of its thelytokous counterparts which unfortunately are scarce in field. We here report the first case of a natural *T*. *dendrolimi* population in China comprising both bisexual wasps and an extremely low proportion of thelytokous wasps. These two forms of *T*. *dendrolimi* are phylogenetically related based on the reconstructions of ITS-2 and COI genes. Also, the phylogenetic results suggested a potentially *Wolbachia*-drived ITS-2 variation. The expression of thelytoky was hardly affected by temperature, which might help control Asian corn borer and *Dendrolimus punctatus*. *Wolbachia* are responsible for current thelytoky according to phylogenetic analyses, antibiotic treatment and introgression experiment. We also present the third case of paternal sex ratio chromosome that restrains the expansion of *Wolbachia*. Moreover, the low frequency of thelytoky may be common in natural populations. Consequently if for biological control it is determined that a thelytokous strain is to be preferred, then large number of field collected females should be set up as isofemale lines, to detect the rare thelytoky.

## Introduction

Augmentative biological control (ABC) concerns periodical inundative release of natural enemies mass-reared in biofactories to promptly control pests^1^. For many years, ABC has been an environmentally and economically successful alternative to chemical pest control^1–3^. It has been estimated that in ABC more than 170 natural enemy species are produced and sold globally for controlling more than 100 pest species on 0.16 million km^2^ (i.e., 0.4% of land under cultivation)^4^. Among these natural enemies, parasitic wasps *Trichogramma* are the most commonly used biocontrol agents against lepidopteran pests throughout the world^5^. Their mass production and inundative release are economically feasible to take a number of most devastating pests under control, such as the Asian corn borer, *Ostrinia furnacalis* Guenée (Lepidoptera: Crambidae)^5^, *Plutella xylostella* (L.) (Lepidoptera: Plutellidae)^6^, and *Tuta absoluta* (Lepidoptera: Gelechiidae)^7,8^.

Different from most other biocontrol agents, these minute egg parasitoids exhibit two modes of reproduction. Typically, they display bisexual reproduction (also referred as arrhenotokous), i.e., the males arise from unfertilized haploid eggs, whereas the females arise from fertilized diploid eggs. But they can also reproduce thelytokously where only diploid females are present. In genus *Trichogramma*, thelytoky can be under the control of the wasps themselves or their endosymbionts. While genetically determined thelytoky was reported only in *Trichogramma cacoeciae*^9^, 15 *Trichogramma* species have been documented to gain thelytoky after being infected with *Wolbachia*^10,11^, the most abundant endosymbionts in arthropods which are famous for their ability to manipulate multiple reproductive phenotypes^12–14^. Though another two microorganisms, *Cardinium* and *Rickettsia*, also cause thelytoky of several arthropod species, none of these cases are within *Trichogramma*^11^. In general, we can distinguish between genetically determined and endosymbionts-induced thelytoky through revertibility. For the former type, thelytoky should be non-revertible which means the reproductive mode is stable and cannot be changed by antibiotic or heat treatment, whereas the latter will produce male or intersex individuals under such conditions^15,16^. Additionally, if thelytoky is genetically determined, the bisexual strains will gain thelytokous reproduction function when alleles of thelytokous strains are introgressed into their genomes^17^. Meanwhile, since endosymbionts are cytoplasmically inherited, the reproductive phenotype induced by them would not change under such introgression.

One important reason we study thelytokous *Trichogramma*, either genetically determined or induced by *Wolbachia*, is to utilize them as potent biocontrol agents. *Trichogramma* wasps are surely preponderant parasitoids of lepidopterans, but an inundative condition is needed to obtain acceptable parasitism effect which makes a high cost^18–20^. Therefore, researchers have been trying to find the best form of *Trichogramma* that could control the pests in lower amount with higher efficiency^21^. And the thelytokous *Trichogramma* may provide us a brilliant future as they offer several advantages: (1) higher population growth rate; (2) less production cost; (3) easier establishment of population; (4) ability to depress host populations to a lower level^22–26^. Unfortunately, this form is only reported in a few *Trichogramma* species in field, and is still lacking in species, like *T*. *dendrolimi* and *T*. *ostriniae* (especially in China), which are predominantly used in biocontrol^18^. Thelytokous *Trichogramma* strains or lines can be artificially obtained by transferring *Wolbachia* inter- or intraspecifically, but in newly infected hosts the expression of induced thelytokous phenotype is generally weak or undetectable, and the infection tends to be lost in most scenarios^27,28^. On the contrary, *Wolbachia* infection and the induced phenotype are stably maintained in natural populations^29,30^. Native strains are also preferred for their better characteristics such as higher tolerance to local environment and higher searching efficiency^31^. Therefore, native thelytokous *Trichogramma* may hold greater biocontrol potential.

In China, *T. dendrolimi* is primarily applied to control Asian corn borer (ACB) which is the most destructive pest of corn occurring in vast majority of Chinese corn-growing areas and cause yield loss ranging from 6 to 9 million tons annually^18^. During 1980s and 1990s, these parasitoid wasps were released annually across 200,000 to 350,000 ha of corn, which increased to 2.3 million ha annually since 2012 in Jilin province, to keep ACB under control^5^. *T. dendrolimi* is also the preponderant parasitoid of *Dendrolimus* spp. (Lepidoptera: Lasiocampidae), the most important conifer defoliators in China whose outbreak is analogized as the ‘fire without smoke'^32^. Moreover, in recent studies, *T. dendrolimi* was identified as the best or promising biological agent of rice stem borer *Chilo suppressalis*^33^, oriental fruit moth *Grapholita molesta* (Lepidoptera: Tortricidae)^34^, box tree pyralid *Cydalima perspectalis* (Lepidoptera: Crambidae)^35^, and oriental armyworm *Mythimna separata* (Lepidoptera: Noctuidae)^36^. *T. dendrolimi* is one of few *Trichogramma* that could be mass reared on big (e.g., *Antheraea pernyi*) and artificial eggs^18,37^. And this wasp species shows a wide distribution in China. It has been found in East (Anhui, Fujian, Jiangsu and Shandong province), South (Guangdong province), North (Beijing municipality and Hebei province), Northeast (Heilongjiang, Jilin and Liaoning province), Northwest (Shaanxi Province) and Southwest (Sichuan province) China (Supplementary Fig. 1). All of these unquestionably make *T. dendrolimi* one of the most successful biocontrol agents.

Previously Grenier *et al*.^27^ reported a successful case of transferring thelytoky-inducing *Wolbachia* into uninfected *T*. *dendrolimi*. But only partial induction of thelytoky was observed in their newly infected wasps. A stable thelytokous *T*. *dendrolimi* line was obtained for the first time in our laboratory by artificially transferring *Wolbachia* from native host *T*. *embryophagum*^10^. However, this line suffers from several drawbacks, such as the longer parasitization cycle, the poor capacity to evaluate the nutritional quality, the higher superparasitism rate and the fewer host eggs oviposited into^20^. We here report, to our knowledge, the first case in China of a natural *T. dendrolimi* population with extremely low proportion of thelytokous individuals. In this study, experiments were conducted to focus primarily on the following questions: (1) Are these thelytokous and bisexual wasps phylogenetically related? (2) Is the thelytoky stable and thus could be applied to biocontrol practice? (3) What is responsible for the thelytoky of *T*. *dendrolimi*? 4) What restrains the expansion of thelytoky in this population? In addition, suggestions of how to detect rare thelytoky in field collected populations were provided. Our finding may offer a powerful option for biocontrol of lepidopteran pests.

## Methods

### Insects

The eggs of *Dendrolimus punctatus* Walker (Lepidoptera: Lasiocampidae) parasitized by *T*. *dendrolimi* were collected on April 16, 2017 from the forest of Chinese red pine in Huanren County, Benxi City of Liaoning Province in China (41°10′N, 125°25′E). Hereafter, the wasps were reared at 25 ± 1 °C, 70 ± 5% relative humidity (RH) with a 16: 8 h light: dark photoperiod on eggs of *Corcyra cephalonica* (Lepidoptera: Pyralidae). And the *C*. *cephalonica* were reared at 26 ± 1 °C, 70 ± 5% RH with a 16: 8 h light: dark photoperiod on maize flour and wheat bran^38^. All the wasps emerged from *D. punctatus* eggs were stored in 70% ethanol after they finished parasitization on *C*. *cephalonica*.

At first glance, the wasps appeared to be bisexual for their seemingly normal sex ratio. Certain females were then found to produce only daughters during isofemale line establishment. These females were separated and kept individually in a glass tube which allowed them to lay unfertilized eggs. More than five generations of continuous reproduction without sex and all female offspring confirmed the existence of thelytoky. In the following contents, the bisexual and thelytokous wasps were designated as Td-HR and TdT-HR strain respectively.

### Genetic backgrounds of Td-HR and TdT-HR strains

The DNA (of 5 individuals randomly selected from all cohorts for each strain) was extracted using Chelex method^39^: briefly, wasps were placed into a 1.5 mL eppendorf tube with 50 μL 5% Chelex-100 and 2.5 μL Proteinase K (20 mg ml^−1^), then incubated for 1 h at 55 °C, and finally followed by 10 min at 99 °C. Nuclear ribosomal DNA (nrDNA) internal transcribed spacer 2 (ITS-2) and two mitochondrial genes cytochrome oxidase I (COI) and cytochrome b (Cytb) were amplified through PCR. These genes were chosen as they appear to represent different modes of evolution. Mitochondrial DNA (mtDNA) was used due to its maternal inheritance, very high mutation rate and lack of recombination^40^, while ITS-2 is a noncoding and therefore rapidly evolving region^41^. Primer pairs used for amplification were: forward 5′-TGTGAACTGCAGGACACATG-3′ and reverse 5′-GTCTTGCCTGCTCTGAG-3′ for ITS-2, forward C1-J-1718 (5′-GGAGGATTTGGAAATTGATTAGTTCC-3′) and reverse C1-N-2191 (5′-CCCGGTAAAATTAAAATATAAACTTC-3′) for COI, and forward CB-J-10933 (5′-TATGTACTACCATGAGGACAAATATC-3′) and reverse CB-N-11367 (5′- ATTACACCTCCTAATTTATTAGGAAT-3′) for Cytb^42,43^. The amplification was performed in a total volume of 50 µL, containing 25 µL 2 × Es Taq Mastermix (CWBIO, Beijing, China), 1 µL each of forward and reverse primers (10 μM), 2 µL DNA template and 21 µL ddH_2_O. Amplification condition for ITS-2 was 3 min at 94 °C, 33 cycles of 40 s at 94 °C, 45 s at 53 °C, and 45 s at 72 °C, followed by 10 min at 72 °C^44^, while for COI and Cytb was 3.5 min at 94 °C, 33 cycles of 35 s at 94 °C, 35 s at 49 °C, and 45 s at 72 °C, followed by 10 min at 72 °C. Resulting PCR products from ITS-2 primers were run on 1% agarose gel and clear target bands were recycled, purified and cloned to pGEM- Teasy vectors (Promega Co., Madison, Wisconsin, USA). Positive ITS-2 clones, COI and Cytb PCR products were sequenced by Invitrogen Trading Co., LTd. (Shanghai, China). The consequent ITS-2 sequences of both Td-HR and TdT-HR strains were directly submitted to the NCBI GenBank database and were assigned accession numbers MG890332 and MG890333 respectively. Only one trimmed COI or Cytb sequence was deposited in the database (MK213321 or MK552378), as the sequencing results from Td-HR and TdT-HR strains were exactly the same. Phylogenetic analyses were conducted based on ITS-2 or COI gene to evaluate the backgrounds of Td-HR and TdT-HR strains. Cytb gene was not used because of lacking enough data in GenBank. For either of the two genes, a data matrix was constructed, including all *T. dendrolimi* reference sequences deposited in GenBank to represent the diversity, 17 other *Trichogramma* species (COI and ITS-2 sequences from the same individuals)^45^ serving as outgroups to evaluate the interspecific distance, and our data. These sequences were aligned by Clustal X version 2.0 (www.clustal.org)^46^ and analyzed using both maximum parsimony (MP) and neighbor joining (NJ) methods with MEGA version 5.0^47,48^, to verify the consistency of the phylogenetic results. Bootstrap analysis was done with 1000 replications to estimate the robustness of the nodes. Evolutionary distances were calculated based on a maximum composite likelihood model. The reference sequences were listed in Supplementary Tables 1 and 2.

### Endosymbionts detection, infection frequency and phylogeny

The *Cardinium*, *Rickettsia* and *Wolbachia* infection were investigated in both Td-HR and TdT-HR strains. Briefly, the DNA of either Td-HR or TdT-HR strain were extracted and then checked for the presence of endosymbionts through PCR with three primer pairs respectively for *Cardinium* (CLO-f1: 5′-GGAACCTTACCTGGGCTAGAATGTATT-3′, CLO-r1: 5′-GCCACTGTCTTCAAGCTCTACCAAC-3′), Rickettsia (R1: 5′-GCTCTTGCAACTTCTATGTT-3′, R2: 5′-CATTGTTCGTCAGGTTGGCG-3′) and *Wolbachia* (81F: 5′-TGGTCCAATAAGTGATGAAGAAAC-3′, 691R: 5′-AAAAATTAAACGCTACTCCA-3′)^49^. PCR amplifications were performed under the conditions described previously and the products were purified, cloned and sequenced as mentioned earlier^49,50^. Fifty individuals were randomly checked each strain. However, only *Wolbachia* were found and only thelytokous individuals were infected. The infection frequency of *Wolbachia* in the original population was then investigated by random detection in 200 individuals stored in the ethanol (see above). Sequence of the *Wolbachia* surface protein gene *wsp* was submitted to NCBI GenBank database and was assigned accession number MG914000. The phylogenetic analysis of *wsp* was carried out as described above. In this analysis, 27 *wsp* gene sequences were used, including our data and 26 reference sequences from NCBI (Supplementary Table 3). To fortify the phylogenetic reconstruction reliability, a MLST typing system was also adopted. PCR amplification was performed for 5 MLST alleles (*gatB*, *coxA*, *hcpA*, *ftsZ*, *fbpA*) following standard protocols and universal primers (https://pubmlst.org/wolbachia/info/protocols.shtml). Nucleotide sequencing data were subsequently deposited in PubMLST database (https://pubmlst.org/wolbachia/). The ST number 486 and id number 1851 were assigned. A matrix comprising our and other 38 unique concatenated MLST sequences retrieved from PubMLST database was used in Maximum Likelihood (ML) analysis to develop the phylogenetic relationship. GTR + G + I nucleotide substitution model was determined as the best fitting choice. ML tree was reconstructed with Mega 5.0 using the following parameters: “No. of Bootstrap Replication” - 1000, “Gaps/Missing Data Treatment” - Use all sites, “No. of Discrete Gamma Categories” - 5. The reference MLST profiles were listed in Supplementary Table 4.

### Antibiotic treatment

To decide whether thelytoky was non-revertible, the females of TdT-HR strain were treated with tetracycline (mixed with 10% honey-water). Five concentrations were used: 0 (control), 0.0001, 0.001, 0.01 and 0.1 mg/ml (C0-C4). Newly emerged females were allowed to feed on tetracycline for 36 h. Then 15 of them were randomly selected from each concentration and supplied individually with approximate 150 (surplus) *C*. *cephalonica* eggs to parasite for 36 h. These females were gently removed after that and the eggs were maintained under 25 ± 1 °C, 70 ± 5% RH condition with a 16: 8 h light: dark photoperiod. The brood size, number of female, male or intersex individulas were counted afterwards. Identification of intersex was attributed to Tulgetske and Stouthamer^16^. Same tetracyline treatments were administrated to mated Td-HR females to serve as a control.

### Introgression experiment

**Figure 1:**
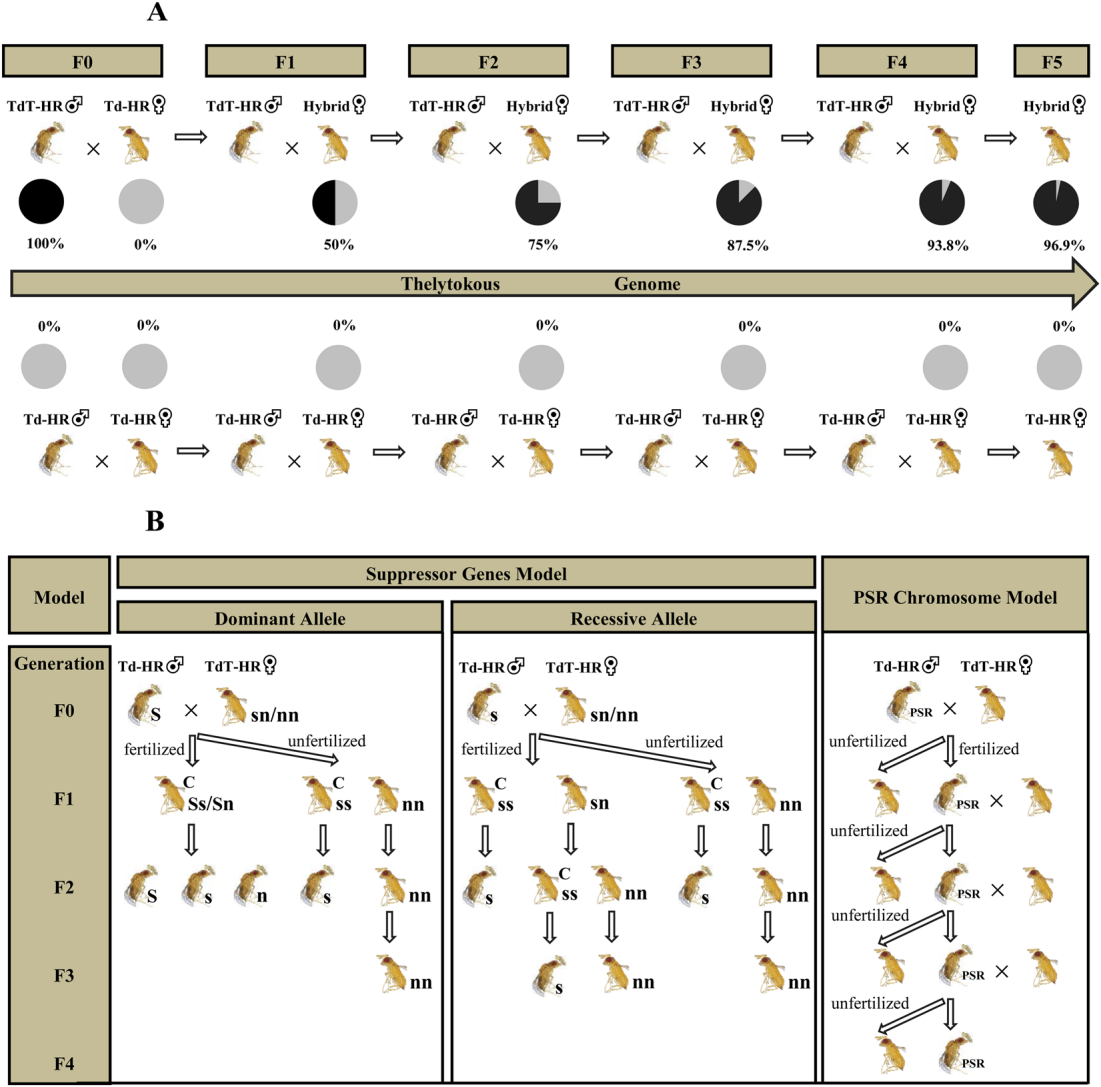
Experiment Design. (**A**) Diagram for Introgression experiment. Black symbols, thelytokous genotypes; Grey symbols, bisexual genotypes. (**B**) Diagram for the detection of suppressor genes or PSR Chromosome. S, dominant suppressor allele; s, recessive suppressor allele; n, non-suppressor allele; C, cured individual.

To test if individuals with a thelytokous genome are able to reproduce thelytokously, alleles of the TdT-HR strain were introgressed into the genomes of Td-HR strain (Fig. 1A). Firstly, 90 females from Td-HR strain were crossed with 90 males from TdT-HR strain (produced by antibiotic treatment) respectively. Then each of these females (F0) was placed in a 4 ml plastic tube with 10% honey-water and supplied with a fresh egg card containing approximately 150 (surplus) *C*. *cephalonica* eggs to oviposit for 24 h. Parasitized eggs blackened within 4 ~ 5 days when wasps pupated. These black eggs were isolated to ensure the offspring (F1) being unmated. Hybrid F1 females were mated again with TdT-HR strain males. This backcross was repeated for 5 generations and the resulting F5 individuals should possess 96.9% of the genome stemming from TdT-HR strain according to the relationship 1 – (0.5)^n ^^17^. In our study, the proportion of hybrid females that produced at least one daughter decreased gradually for the possible decayed sexual traits in thelytokous strain or the diploid males which are usually stertile or sire inviable or sterile triploid females^51–54^. Consequently, the number of hybrid lines used for reproduction mode analysis decreased from 90 to 33 in F1 generation, to 17 in F2, to 8 in F3, to 7 in F4 and 4 in F5. The reproduction mode of hybrid females was tested every generation (averaging 12 virgins selected for each hybrid line) by supplying them with *C*. *cephalonica* eggs. All female offspring indicated the mode is thelytoky, whereas all male offspring indicated a arrhenotokyous reproduction mode. The control was conducted by performing a continuous cross between bisexual Td-HR females and males.

### Factors restraining expansion of thelytoky

From the results of above experiments, it can be concluded that *Wolbachia* induce the thelytoky of *T*. *dendrolimi* (see Results and Discussion for further details). And also, because the infection only existed in thelytokous wasps (see Endosymbionts detection, infection frequency and phylogeny), our fourth question, i.e., what restrains the expansion of thelytoky, was thereby simplified to what keeps the frequency of *Wolbachia* low? Stouthamer *et al*.^55^ proposed three factors that could serve to the restricted spread of *Wolbachia*, including: (1) inefficient transmission of *Wolbachia* which causes some offspring of infected females to lose infection. (2) suppressor genes based on the conflict between nuclear and cytoplasmic genes. Since *Wolbachia* are cytoplasmic inherited, they cannot benefit from the male fitness and favor a female biased population. In the meantime, males would try to suppress the transmission of *Wolbachia*. Several instances have been reported where suppressors act in two ways (i.e., by killing the bacteria or by negating the effects from them) against cytoplasmic sex-ratio distorters^56–58^. (3) presence of paternal sex ratio (PSR) chromosome which acts by destroying the paternal set of chromosomes after fertilization, with the exception of itself. Therefore the resulting sons only carry maternal and PSR chromosomes^55,59,60^. With regard to the first factor, if *Wolbachia* do transfer inefficiently, the expression level of thelytokous phenotype in hosts will decrease gradually with increasing generations, especially under adverse environments. Apparently, this is exactly what we need to determine in the second question, i.e., is the thelytoky stable? To answer this question, a successive observation on sex ratio was carried out for thelytokous strain at four temperatures: 17, 21, 25 and 29 °C. The 33 °C was not performed because it caused a substantial number of deaths. Thirty five TdT-HR wasps were individually maintained at each rearing temperature in a growth chamber (Panasonic, MLR-352H-PC, Osaka, Japan) for four generations. For each generation, superfluous *C*. *cephalonica* eggs were supplied. The number of emerged female, male or intersex wasps was counted, then average and total (i.e., compared to total emerged offspring) rates of male or intersex were calculated. Another experiment for differentiating the latter two factors was designed according to Stouthamer *et al*.^55^ (see also Fig. 1B). Sixty cross lines (F0) between bisexual Td-HR males and thelytokous TdT-HR females were established, 15 (if possible) resulting virgin daughters (F1) from each cross line were selected randomly to produce F2 offspring (granddaughters). The virgin production was continued one more generation by selecting 30 granddaughters (2 granddaughters × 15 daughter lines) each cross line. Virgin females were obtained as described in Introgression section. Suppose that suppressor genes do present, a male carrying a suppressor allele will produce cured daughters (dominant allele or both male and female carrying a recessive allele), or cured granddaughters (when male with a recessive allele)^55^. In light of this, males would occur in F2 or F3 generation. Under PSR chromosome model, however, males would occur in F1 and usually with a high proportion. Additionally, if such high male proportion occurred, the males from high male biased lines would be crossed with thelytokous females three more generations.

### Statistical analysis

The possible effects of tetracycline treatment on brood size, sex ratio (males / total emerged wasps) were evaluated by using generalized linear model (GLM), with tetracycline treatment as the explanatory variable, while brood size or sex ratio was modeled as response variable based on a Poisson distribution or a binomial logit distribution. To identify if number of TdT-HR male or intersex offspring changed with different antibiotic concentrations, generalized linear mixed model (GLMM) was perfromed based on Poisson distribution. Tetracycline concentration was used as explanatory variable, male or intersex offspring number as the response variable, and brood size as the random factor.

All statistical analyses were performed in R software (version 3.4.3)^61^.

## Results

### Genetic backgrounds of Td-HR and TdT-HR strains

**Figure 2:**
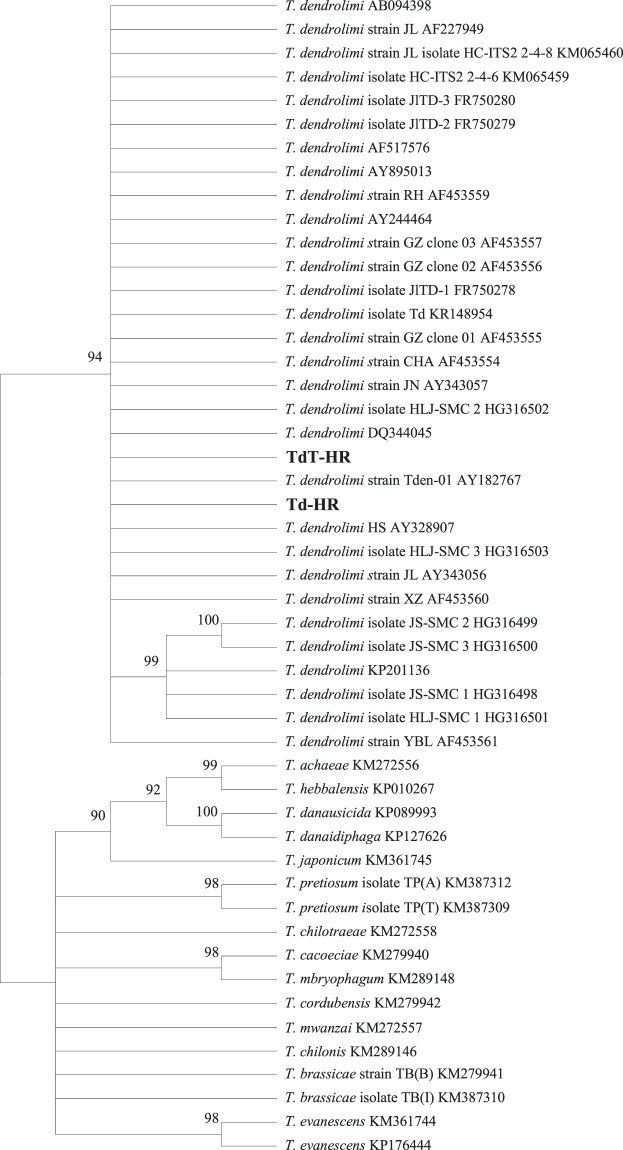
Phylogenetic Neighbor Joining tree of *Trichogramma* species based on ITS-2 sequences. Accession number follows the taxon name. Designations in bold are the sequences of bisexual and thelytokous *T*. *dendrolimi* strains in this study. Values above the branches are the percentages of bootstrap support estimated from 1000 replicates.

**Figure 3:**
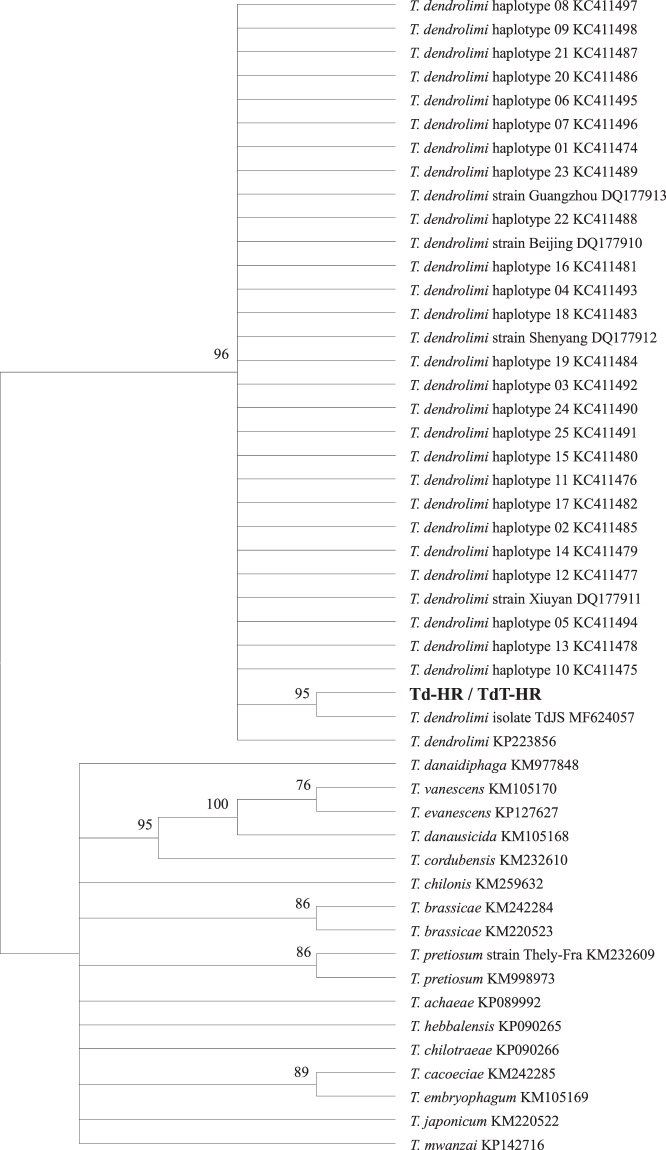
Phylogenetic Neighbor Joining tree of *Trichogramma* species based on COI sequences. Accession number follows the taxon name. Designations in bold are the sequences of bisexual and thelytokous *T*. *dendrolimi* strains in this study. Values above the branches are the percentages of bootstrap support estimated from 1000 replicates.

ITS-2 gene showed a relative high variation. The distance based on ITS-2 gene between TdT-HR and Td-HR was 0.015 (1.55% sequence divergence), higher than that between TdT-HR and most other *T*. *dendrolimi* strains or isolates (0.003–0.012). Not a single base difference was found in COI or Cytb sequences between Td-HR and TdT-HR strains. Mean intraspecific and interspecific distances were 0.043 and 0.276 for ITS-2, whereas were 0.006 and 0.065 for COI. The reconstructed phylogenetic relationship using ITS-2 or COI sequences is presented as a phylogram in Figs. 2 or 3. As the trees inferred from neighbor joining and maximum parsimony methods were similar, only the former one is displayed (same for *Wolbachia* phylogeny). ITS-2 tree indicates that both Td-HR and TdT-HR strains belong to a widely unresolved monophyletic polytomy (94% bootstrap support) of highly similar *T*. *dendrolimi* isolates and strains from various origins, including those from Germany, Japan and different regions of China (Supplementary Table 1). The clade appearing as highly homogenous *T*. *dendrolimi* genetic pool is clearly divergent from all the other *Trichogramma* species used in this study (Fig. 2). A similar phylogenetic COI-based tree further indicates that our strains and isolate TdJS are sister taxa (95% bootstrap support). Interestingly, the isolate TdJS is from Jilin province, adjacent to our sampling location on the east by city Ji’an and north by city Tonghua, implying that these two populations may have same geographic origin.

### *Wolbachia* infection frequency and phylogeny

**Figure 4:**
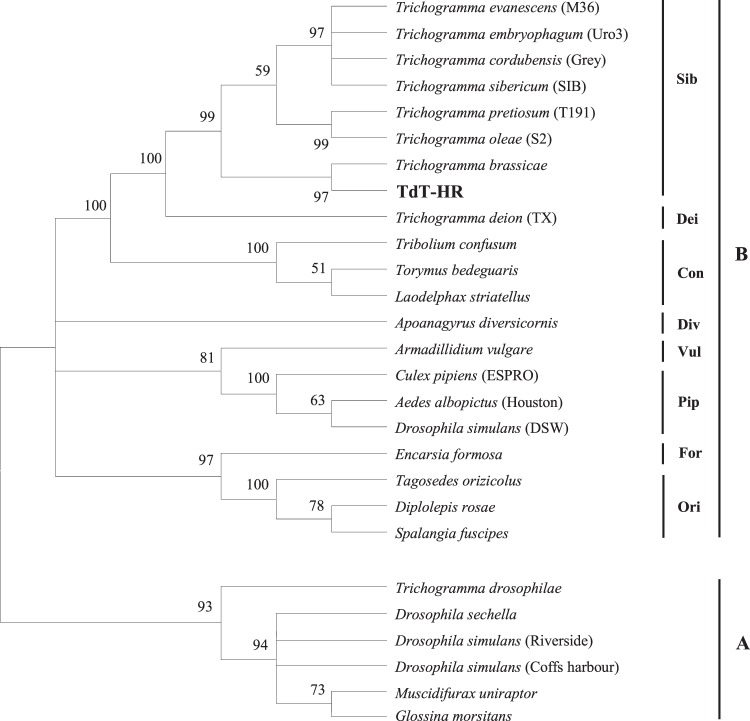
Phylogenetic Neighbor Joining tree of *Wolbachia* based on *wsp* sequences. Designation in bold is the sequence from thelytokous *T*. *dendrolimi* in current study. Values above the branches are the percentages of bootstrap support estimated from 1000 replicates.

**Figure 5:**
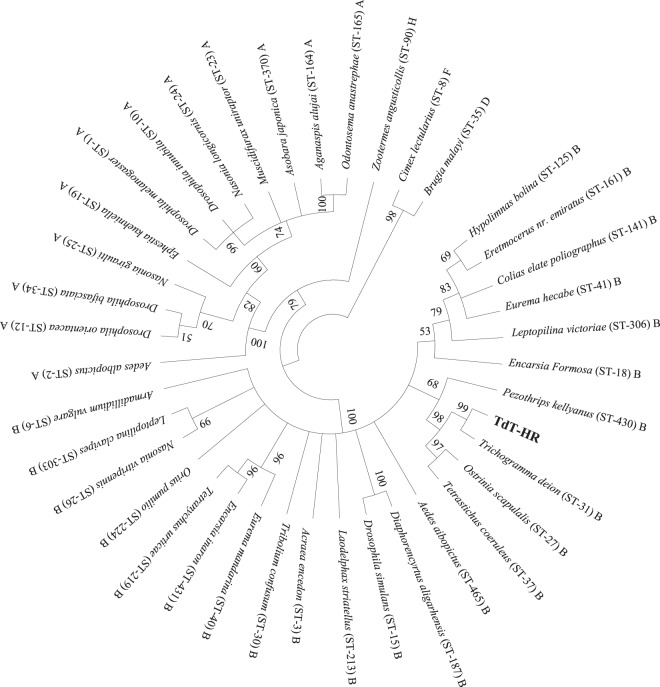
Phylogenetic Maximum Likelihood tree of *Wolbachia* based on 5 MLST alleles (*gatB*, *coxA*, *hcpA*, *ftsZ*, *fbpA*). ST number and supergroup follow the taxon name. Designation in bold is the *Wolbachia* strain from our thelytokous *T*. *dendrolimi*. Values above the branches are the percentages of bootstrap support estimated from 1000 replicates.

The detection of endosymbionts was performed for both Td-HR and TdT-HR strains, but only *Wolbachia* was found and only thelytokous wasps were infected. Among 200 examined wasps, two females (1%) were infected by *Wolbachia*. The phylogenetic *wsp*-based tree (Fig. 4) is obviously divided into two supergroups (A and B) which is consistent with previous studies^50,62^. According to the grouping criterion (2.5% divergence) by Zhou *et al*.^50^, *wsp* sequences of supergroup B used in this study were subdivided into eight groups. Our *Wolbachia* strain showed 0.6 to 2.1% *wsp* sequence differences from other *Wolbachia* strains in Sib group and thus fell within this group. Similarly, ML phylogeny of *Wolbachia* based on 5 concatenated MLST alleles (2,079 bp after aligned and trimmed) from our data and different supergroups (A, B, D, F and H) from PubMLST reveals an incidence of *Wolbachia* in TdT-HR wasps belonging to supergroup B and a monophyletic higher relatedness to *T. deion* (Fig. 5).

### Antibiotic treatment

**Figure 6:**
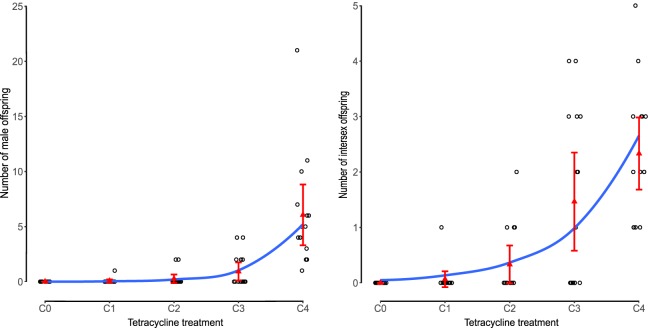
The number of male or intersex offspring of thelytokous TdT-HR strain females treated with different concentrations of tetracycline. C0 is the no tetracycline treatment control, C1 to C4 represent 0.0001, 0.001, 0.01 and 0.1 mg/ml tetracycline respectively.

To investigate whether thelytoky is unrevertible, five concentrations of tetracycline (C0-C4) were administrated to the females of TdT-HR strain. Statistical analyses showed that both male and intersex offspring gradually increased with higher tetracycline concentrations, ranging from 0 to 91 (11.73%) for male (GLMM, χ^2^ = 76.09, d.f. = 1, *P < *0.001) and to 35 (4.51%) for intersex (GLMM, χ^2^ = 46.904, d.f. = 1, *P < *0.001) (Fig. 6 and Supplementary Fig. 2). Same tetracycline treatment was performed on Td-HR strain to exclude the possible effects of tetracycline. But no intersex individuals were found and no significant differences were observed in sex ratio (GLM, χ^2^ = 1.8361, d.f. = 4, *P* = 0.7659) or brood size (GLM, χ^2^ = 1.4848, d.f. = 4, *P* = 0.8293) among tetracycline-treated and untreated groups.

### Introgression

If the thelytoky is genetically determined, the bisexual Td-HR wasps should gain thelytokous reproduction function after alleles of thelytokous TdT-HR wasps are introgressed into their genomes. A continuous introgression carried out between TdT-HR males and Td-HR females showed that the hybrid virgin females in every generation remained arrhenotokous (Table 1), proving that the genome is not responsible for the thelytoky of *T. dendrolimi*.

**Table 1:** The offspring produced by hybrid virgin females of different introgression generations.

Cross	F1	F2	F3	F4	F5
TdT-HR♂ × Td-HR♀	all ♂ n = 350	all ♂ n = 171	all ♂ n = 89	all ♂ n = 72	all ♂ n = 48
Td-HR♂ × Td-HR♀(control)	all ♂ n = 141	all ♂ n = 130	all ♂ n = 150	all ♂ n = 150	all ♂ n = 136

Sample sizes (n) are given for each generation.

### Factors restraining expansion of thelytoky

Of three possible factors that may restrict the spread of *Wolbachia*, inefficient transmission results in a high proportion of abnormal (male or intersex) offspring. But this was not found in TdT-HR strain. As shown in Table 2, males and intersex individuals were rare or absent in each generation under low (17 and 21 °C), moderate (25 °C) and high (29 °C) temperatures, demonstrating the stable transmission of *Wolbachia*. Assuming that suppressor genes are present, the offspring of cured virgin daughters (F1) or granddaughters (F2) should be all male (See Fig. 1B). However, no such daughters or granddaughters were found (Supplementary Table 5). Instead, our results accorded well with the model of PSR chromosome. Among 54 hybrid lines established successfully (i.e., the F0 female produced at least one offspring), nine (16.67%) showed high male proportion in F1 generation (Supplementary Table 5). Males from these male biased lines were crossed with TdT-HR females for three consecutive generations. All resulting hybrid lines produced mostly male offspring (Supplementary Table 6).

**Table 2:** Average and total rates of male or intersex wasps under different temperature treatments.

Temperature	F1				F2				F3				F4			
	♂(%)		intersex(%)		♂(%)		intersex(%)		♂(%)		intersex(%)		♂(%)		intersex(%)	
	avg	total	avg	total	avg	total	avg	total	avg	total	avg	total	avg	total	avg	total
17	0.20	0.22	0	0	0	0	0	0	0	0	0	0	0.15	0.20	0	0
21	0	0	1.47	1.57	0	0	0	0	0	0	0.43	1.49	0	0	0	0
25	0	0	0	0	0	0	0	0	0	0	0	0	0	0	0	0
29	0	0	0	0	1.25	2.20	0	0	0.81	1.18	0	0	1.25	1.40	0	0

## Discussion

*Trichogramma dendrolimi* is one of the most important biocontrol agents widely used in agriculture and forestry production for its high parasitism rate, adaption to multiple lepidopteran pests, wide distribution, and ability to be mass reared on big or artificial eggs^18,32–37^. But as mentioned earlier, an acceptable parasitism effect requires an inundative condition which makes a high cost. A good solution to this is the practice of releasing its thelytokous counterparts. We here report the first thelytokous *T*. *dendrolimi* strain in China which co-occurs with its bisexual form in a natural population.

We first analyzed the phylogenetic backgrounds of the two strains. ITS-2 gene has widely proven to be a powerful tool in distinguishing among closely related *Trichogramma* species^42,63–66^. Its usefulness for species identification in *Trichogramma* is because: (1) smaller divergence within species than between species; (2) morphologically distinct cryptic species can be discriminated by sequence differences^42^. The conservative sequence variation is related to concerted evolution which results in homogenization of variable repeats and produces a mostly uniform sequence within individuals of a population and hence among panmictic populations within species^67–69^. Phylogenetic analysis based on ITS-2 gene apparently supported that our two strains are part of the highly homogeneous *T. dendrolimi* genetic pool and can be assumed to have similar genetic backgrounds, as suggested by Kishani *et al*.^44,70^. Such relationship was further substantiated by similar COI-based reconstruction and by undifferentiated COI and Cytb sequences between two strains. These two mitochondrial genes have also been proved to provide abundant phylogenetic information in *Trichogramma*^45,71,72^. Worthy of note is that: 1) ITS-2 gene in *T*. *dendrolimi* appears to represent a higher intraspecific variation and a faster evolution (i.e., longer interspecific distances) than COI; 2) Td-HR is not phylogenetically closest to TdT-HR based on ITS-2 but COI. The higher intraspecific variation of ITS-2 seems unusual since COI generally has a greater nucleotide diversity within species^41,73–75^. Several alternative hypotheses could be applied to the polymorphous ITS-2. Firstly, it could be simply interpreted as the result of mutation accumulation (e.g., *Taraxacum offcinale*)^76^. Secondly, the gene flow between races, ecotypes or subspecies of a species that have distinct histories could serve to the mixing of differentiated ITS-2 sequences, postponing the homogenization^73^. The final hypothesis is the lack of gene flow causing retention of different ITS-2 morphisms^77^.

The mutation accumulation, if does exist, one would then expect random mutations of ITS-2 gene. However, no differences were found in sequences of cloned ITS-2 gene from either bisexual or thelytokous wasps. Gene flow requires different clusters with distinct historic backgrounds. This overtly contradicts the undifferentiated COI and Cytb sequences between Td-HR and TdT-HR strains. The lack of gene flow between thelytokous and bisexual wasps is more plausible. If the polymorphism in ITS-2 sequences is present in ancestral population, the subsequent *Wolbachia* infection causing thelytoky (see below), male mating preference for bisexual females^78,79^, and PSR chromosome eliminating paternal genome after inseminating thelytokous wasps (see below) would reduce or prevent the gene flow, and hence sharpen the different patterns of ITS-2 between bisexual and thelytokous wasps. It is also possible that the polymorphism happens after *Wolbachia* infection. In this case, *Wolbachia* is responsible for nonrandom mutation of ITS-2 in thelytokous strain. Either way, *Wolbachia* seem to play a role in driving the variation of ITS-2. In addition, the phylogenetic COI-based reconstruction revealed that our population represents a sister taxon to the one from Jilin province that is adjacent to our sampling location. This may imply a wider distribution of *Wolbachia*-infected thelytokous *T*. *dendrolimi*.

Of three endosymbionts causing thelytoky of arthropods, the wasps of TdT-HR strain were only infected by *Wolbachia*. Phylogenetic analyses indicated that this *Wolbachia* strain belongs to Sib group (in supergroup B) which has proven to be able to induce thelytoky in *Trichogramma*. To date, 16 *Wolbachia* supergroups (A–F and H–Q) have been established^80^. Nevertheless, the thelytoky-inducing strains (with firm demonstrations) are reported only in two supergroups: A and B^11,81^. In supergroup A, two groups (i.e., Dro and Uni) cause thelytoky of four species, including *Telenomus nawai*, *Muscidifurax uniraptor*, and two *Aphytis* species^62,82–84^. Meanwhile, three such groups (i.e., Dei, Div and Sib) are found in supergroup B: Dei and Sib are responsible for the thelytoky of 9 *Trichogramma* species, including *T. brassicae*, *T. chilonis*, *T. cordubensis, T. deion*, *T. dendrolimi*, *T. embryophagum*, *T. kaykai*, *T. oleae* and *T. pretiosum*, while Div only induces thelytoky in *Apoanagyrus diversicornis*^10,28,62,85–90^. Since for many host species the *Wolbachia* group information was not supplied (e.g., *Bryobia praetiosa*)^91^, the thelytokous groups listed here could be incomplete. It is interesting to note that the group Dei-induced thelytokous *T*. *dendrolimi* was demonstrated in our previous study by transferring *Wolbachia* from *T*. *embryophagum*^10^. This suggests that the reproduction of a host can be manipulated by different *Wolbachia*.

Both antibiotic treatment and introgression experiment proved that current thelytoky is a result of microorganism infection instead of a genetically determined mechanism. Furthermore, the expression of thelytokous phenotype significantly weakened when tetracycline concentration was increased. It may suggest that the microorganism(s) inside the wasps was responding to the change of antibiotic when the effects of tetracycline on *T*. *dendrolimi* were already ruled out. Previous studies have reported a positive correlation between thelytokous phenotype and *Wolbachia* titer: Wang *et al*.^92^ showed a decreased female offspring proportion of thelytokous *Encarsia Formosa* with lower *Wolbachia* titer; Ma *et al*.^93^ pointed out that the males of thelytokous *Asobara japonica* increased with the decrease of *Wolbachia* titer caused by Rifampicin. Indeed, through quantitative PCR, we were able to prove that *Wolbachia* titer significantly decreased with higher tetracycline concentrations (or with weakened thelytokous phenotype) (see Supplementary Fig. 3).

The similar backgrounds, endosymbiont-induced thelytoky, *Wolbachia* infection, thelytoky-inducing group Sib, and positive correlation between *Wolbachia* titer and thelytokous phenotype allowed us to reasonably reach a conclusion that *Wolbachia* do induce the thelytoky of TdT-HR wasps. And we could thereby proceed to the next question: why the *Wolbachia* infection rate was so low in this population? Generally, a fixed *Wolbachia* infection is expected, i.e., all individuals (or nearly 100%) are female^94,95^. But the low infection level appears to be not rare: the rate was 0.9%, 4.5% and 4–26% for *T. evanescens*, *T*. *turkestanica* and *T*. *kaykai* Pinto & Stouthamer, respectively^31,55^. Among the factors that serve to the restricted spread of *Wolbachia*, the inefficient transmission was firstly examined. We failed to find a high, but an extremely low rate of abnormal individuals in TdT-HR strain at low, moderate or high temperature. This suggested a stable transmission of *Wolbachia*, which is the prerequisite for us to employ this strain in biocontrol practice. In Northeast China, ACB generally has two generations per year corresponding to two different environmental temperatures^18^. In first generation when it is around 25 °C^20^, ABC larvae emerge from eggs on whorl-stage corn and cause seriously direct damages. But a much greater indirect damage occurs in the second generation when corn reaches silking or pollen-shedding stage at much higher temperatures^96^, because the feeding activity from larvae can cause ear and kernel rot which substantially raise the risk of grains contamination by mycotoxins^19^. Using TdT-HR strain to control ABC might be practicable on account of its stable thelytokous phenotype under different temperatures. It also seems likely that this strain displays a good control of *Dendrolimus punctatus* for being a native parasitoid of this pest species.

Based on models attributed to Stouthamer *et al*.^55^, we were able to detect and distinguish between suppressor genes and PSR chromosome. Our results evidently supported the latter. Thus far, the selfish chromosome PSR has only been described in two wasps, *Nasonia vitripennis* and *Trichograma kaykai* Pinto & Stouthamer^97^. We here provide *T*. *dendrolimi* as the third case. Additionally, it is important to note the extremely low *Wolbachia* infection caused by PSR chromosome. As in such a case the corresponding thelytokous wasps would be too rare to be discriminated from normal bisexual wasps. Consequently, the frequency of *Wolbachia*-induced thelytoky in nature would be misestimated, or worse, we cannot give an objective assessment of the interaction between *Wolbachia* and their hosts. In current study, a description of how we “accidentally” found the thelytokous strain was provided, that is, by setting up isofemale lines. We herein also recommend that researchers firstly establish the isofemale lines of parasitoid wasps (or other haplo-diploid species) from fields before detecting *Wolbachia* infection for the fact of rare thelytoky.

For a long time, thelytokous *Trichogramma* have been considered to enhance the efficacy of biocontrol. But their application is prevented by the fact of being scarce in nature. Efforts have been made to artificially create thelytokous *Trichogramma* wasps^27^, and the common way is to transfer *Wolbachia* from native into foreign hosts. So far, two methods have now been mainly adopted: microinjection and host sharing. The former is more general, whereas the latter has only been used in parasitoid wasps^10,27,28,98–100^. Successful *Wolbachia* transinfection are reported notwithstanding, compared to native hosts, *Wolbachia* in the foreign ones universally induce a weaker or undetectable phenotype and usually cannot be maintained stably, even if the transinfection is between conspecifics. The transmission rate of thelytoky-inducing *Wolbachia* decreased to 0% at seventh generation in the new host *Drosophila simulans* (*Muscidifurax uniraptor* as the donor)^98^, and similar results were found in the recipient hosts *T*. *kaykai, T*. *deion* and *T*. *atopovirilia* (Intra- or interspecific *Trichogramma* as the donors)^28^; a low vertical transmission rate of *Wolbachia* causing cytoplasmic incompatibility was shown in the recipient host *Ostrinia scapulalis* (*Ephestia kuehniella* as the donor)^100^. In contrast, the *Wolbachia* infection and induced phenotype in natural strains are maintained stably in general^29,30^, possibly for the long-term co-adaptation between *Wolbachia* and their native hosts.

Finally, we are able to answer the questions addressed in Introduction: yes, thelytokous and bisexual wasps are phylogenetically related; and yes, the thelytoky is stable; *Wolbachia* is responsible for current thelytoky and PSR chromosome restrains their expansion. However, we still lack the comprehensive studies and integrate evaluation to determine the biocontrol potential of our thelytokous strain before it can be used in practice. And our further studies will focus on this aspect by evaluating indicators like body size, vagility^101^, decision-making^20^, and adaptive capacity to the long-term storage^90^.

## Supplementary information

supplementary information

## Data Availability

All data included in this study are available upon reasonable request by contact with the corresponding author.
