# Supplementary material for: Co-occurrence of thelytokous and bisexual Trichogramma dendrolimi Matsumura (Hymenoptera: Trichogrammatidae) in a natural population
Source: Sci Rep. 2019 Nov 25;9:17480. doi: 10.1038/s41598-019-53992-8 (PMC6877646; doi:10.1038/s41598-019-53992-8)
Supplement: Supplementary file 1 — supplementary information [file 41598_2019_53992_MOESM1_ESM.pdf]

Co-occurrence of thelytokous and bisexual *Trichogramma dendrolimi* Matsumura (Hymenoptera: Trichogrammatidae) in a natural population

Short running title: First case of thelytokous *T. dendrolimi* in China

Quan-quan Liu[1,†], Jin-cheng Zhou[1,†], Chen Zhang[1], Su-fang Ning[1], Li-jia Duan[1], Hui Dong[1,]*

* *Correspondence to: Hui Dong, College of Plant Protection, Shenyang Agricultural University, Shenyang, Liaoning, 110866, China*
E-mail: biocontrol@163.com

[†] *These authors contributed equally to this work*

[1] *College of Plant Protection, Shenyang Agricultural University, Shenyang, Liaoning, China*

Supplementary Table 1. Reference sequences for ITS2 based phylogenetic analysis.

Trichogramma spp.	Strain or isolate	Origin	Accession NO.
T. dendrolimi	isolate Fukuyama	Fukuyama in Hiroshima, Japan	AB094398
	isolate HC-ITS2 2-4-6	South Korea (37.53 N 128.20 E)	KM065459
	isolate HC-ITS2 2-4-8	South Korea (37.53 N 128.20 E)	KM065460
	isolate HLJ-SMC 1	Heilongjiang Province, China	HG316501
	isolate HLJ-SMC 2	Heilongjiang Province, China	HG316502
	isolate HLJ-SMC 3	Heilongjiang Province, China	HG316503
	isolate JlTD-1	Changchun in Jilin Province, China	FR750278
	isolate JlTD-2	Changchun in Jilin Province, China	FR750279
	isolate JlTD-3	Changchun in Jilin Province, China	FR750280
	isolate JS-SMC1	Jiangsu Province, China	HG316498
	isolate JS-SMC 2	Jiangsu Province, China	HG316499
	isolate JS-SMC 3	Jiangsu Province, China	HG316500
	isolate Td	Nanjing in Jiangsu Province, China (32°3’0”N, 118°46’60”E)	KR148954
	strain CHA	Chang’ an in Shaanxi Province, China	AF453554
	strain GZ clone 01	Guangzhou in Guangdong Province, China	AF453555
	strain GZ clone 02	Guangzhou in Guangdong Province, China	AF453556
	strain GZ clone 03	Guangzhou in Guangdong Province, China	AF453557
	strain HS	Hengshui in Hebei Province, China	AY328907
	strain JL	Changchun in Jilin Province, China	AF227949
	strain JL	Jilin Province, China	AY343056
	strain JN	Ju'nan in Shandong Province, China	AY343057
	strain RH	Renhe in Jilin Province, China	AF453559
	strain Shenzhen	Shenzhen in Guangdong Province, China	AY244464
	strain Tden-01	Germany	AY182767
	strain XZ	Xuzhou in Jiangsu Province, China	AF453560
	strain YBL	Yabuli in Heilongjiang Province, China	AF453561
	—	Japan	AY895013
	—	—	AF517576
	—	—	DQ344045
	—	Germany	KP201136
T. achaeae	—	Gujarat Province, India	KP089992
T. brassicae	—	Canada	KM242284
T. brassicae	—	Italy	KM220523
T. cacoeciae	—	France	KM242285
T. chilonis	—	India	KM259632
T. chilotraeae	—	India	KP090266
T. cordubensis	—	France	KM232610
T. danaidiphaga	—	India	KM977848
T. danausicida	—	India	KM105168
T. embryophagum	—	Germany	KM105169

T. evanescens	—	France	KP127627
T. hebbalensis	—	Karnataka Province, India	KP090265
T. japonicum	—	India	KM220522
T. mwanzai	—	Kenya	KP142716
T. pretiosum	strain Thely-Fra	France	KM232609
T. pretiosum	—	Germany	KM998973
T. vanescens	—	France	KM105170

Supplementary Table 2. Reference sequences for COI based phylogenetic analysis.

Trichogramma spp.	Strain or isolate	Origin	Accession NO.
T. dendrolimi	haplotype 01	—	KC411474
	haplotype 02	—	KC411485
	haplotype 03	—	KC411492
	haplotype 04	—	KC411493
	haplotype 05	—	KC411494
	haplotype 06	—	KC411495
	haplotype 07	—	KC411496
	haplotype 08	—	KC411497
	haplotype 09	—	KC411498
	haplotype 10	—	KC411475
	haplotype 11	—	KC411476
	haplotype 12	—	KC411477
	haplotype 13	—	KC411478
	haplotype 14	—	KC411479
	haplotype 15	—	KC411480
	haplotype 16	—	KC411481
	haplotype 17	—	KC411482
	haplotype 18	—	KC411483
	haplotype 19	—	KC411484
	haplotype 20	—	KC411486
	haplotype 21	—	KC411487
	haplotype 22	—	KC411488
	haplotype 23	—	KC411489
	haplotype 24	—	KC411490
	haplotype 25	—	KC411491
	isolate TdJS	Jilin Province, China	MF624057
	strain Xiuyan	Xiuyan in Liaoning Province, China	DQ177911
	strain Beijing	Beijing, China	DQ177910

	strain Shenyang	Shenyang in Liaoning Province, China	DQ177912
	strain Guangzhou	Guangzhou in Guangdong Province, China	DQ177913
	—	Germany	KP223856
T. achaeae	—	Gujarat Province, India	KP089992
T. brassicae	—	Canada	KM242284
T. brassicae	—	Italy	KM220523
T. cacoeciae	—	France	KM242285
T. chilonis	—	India	KM259632
T. chilotraeae	—	India	KP090266
T. cordubensis	—	France	KM232610
T. danaidiphaga	—	India	KM977848
T. danausicida	—	India	KM105168
T. embryophagum	—	Germany	KM105169
T. evanescens	—	France	KP127627
T. hebbalensis	—	Karnataka Province, India	KP090265
T. japonicum	—	India	KM220522
T. mwanzai	—	Kenya	KP142716
T. pretiosum	strain Thely-Fra	France	KM232609
T. pretiosum	—	Germany	KM998973
T. vanescens	—	France	KM105170

Supplementary Table 3. Reference *wsp* sequences for phylogenetic analysis of *Wolbachia*.

Supergroup	Group	*Wolbachia* host species	*Wolbachia* strain	Accession NO.
A	Dro	*Trichogramma drosophilae*	*w*Dro	AF071910
	Ha	*Drosophila sechella*	*w*Ha	AF020073
	Mel	*Drosophila simulans* (Coffs	*w*Cof	AF020067
	Mors	*Glossina morsitans*	*w*Mors	AF020079
	Riv	*Drosophila simulans* (Riverside)	*w*Ri	AF020070
	Uni	*Muscidifurax uniraptor*	*w*Uni	AF020071
B	Con	*Laodelphax striatellus*	*w*Stri	AF020080
		Torymus bedeguaris	*w*Bed	AF071915
		Tribolium confusum	*w*Con	AF020083
	Dei	*Trichogramma deion* (TX)	*w*Dei	AF020084
	Div	*Apoanagyrus diversicornis*	*w*Div	AF071916
	For	*Encarsia formosa*	*w*For	AF071918
	Ori	*Diplolepis rosae*	*w*Ros	AF071922

		Spalangia fuscipes	*w*Fu	AF071921
		Tagosedes orizicolus	*w*Ori	AF020085
	Pip	*Aedes albopictus* (Houston)	*w*AlbB	AF020059
		Culex pipiens (ESPRO)	*w*Pip	AF020061
		Drosophila simulans (DSW)	*w*Ma	AF020069
	Sib	*Trichogramma brassicae*	*w*Na	JX131628
		Trichogramma cordubensis (Grey)	Grey	AF245164
		Trichogramma embryophagum	Uro3	AF245165
		Trichogramma evanescens (M36)	M36	AF245167
		Trichogramma oleae (S2)	S2	AF245166
		Trichogramma pretiosum (T191)	T191	AF245163
		Trichogramma sibericum (SIB)	*w*Sib	AF071923
	Vul	*Armadillidium vulgare*	*w*Vul	AF071917

Supplementary Table 4. Reference MLST gene sequences for phylogenetic analysis of *Wolbachia*

Supergroup	*Wolbachia* host species	MLST alleles					ST	id
		gatB	*cox*	*hcp*	*ftsZ*	*fbpA*		
A	*Aedes albopictus*	3	2	2	10	3	2	12
	Drosophila melanogaster	1	1	1	1	1	1	1
	Drosophila orientacea	10	10	11	14	11	12	8
	Ephestia kuehniella	7	6	7	3	8	19	13
	Nasonia giraulti	11	17	18	3	17	25	17
	Nasonia longicornis	2	1	1	3	1	24	16
	Drosophila bifasciata	14	15	16	13	15	34	5
	Drosophila innubila	1	1	1	3	2	10	6
	Aganaspis alujai	54	52	62	82	62	164	96
	Asobara japonica	87	111	103	70	186	370	29
	Muscidifurax uniraptor	8	7	8	6	1	23	15
	Odontosema anastrephae	54	52	62	3	164	165	25
B	*Aedes aibopictus*	247	229	166	210	27	465	17
	Colias elate poliographus	100	14	40	73	4	141	19
	Drosophila simulans	5	4	5	4	6	15	26
	Encarsia inaron	233	38	3	73	4	431	16
	Eretmocerus nr emiratus	105	14	3	73	4	161	72
	Laodelphax striatellus	106	11	13	105	162	213	31
	Leptopilina victoriae	195	182	206	22	4	306	50
	Nasonia vitripennis	9	8	9	7	9	26	34

	Orius pumilio	139	125	147	111	200	224	32
	Pezothrips kellyanus	9	14	106	7	89	430	16
	Tetranychus urticae	9	38	143	23	4	219	31
	Tribolium confusum	6	5	6	18	7	30	20
	Eurema hecabe	39	14	40	36	4	41	29
	Eurema mandarina	38	38	29	35	42	40	20
	Armadillidium vulgare	13	13	14	9	13	6	28
	Acraea encedon	9	11	12	11	12	3	22
	Hypolimnas bolina	4	14	40	73	4	125	40
	Ostrinia scapulalis	9	9	10	8	10	27	32
	Diaphorencyrtus aligarhensis	125	4	74	4	6	187	28
	Encarsia formosa	17	18	20	15	18	18	33
	Leptopilina clavipes	9	126	148	7	201	303	34
	Tetrastichus coeruleus	9	9	6	8	10	37	29
	Trichogramma deion	21	22	25	19	21	31	35
D	*Brugia malayi*	28	29	33	26	30	35	37
F	*Cimex lectularius*	26	27	31	24	28	8	36
H	*Zootermes angusticollis*	64	54	71	57	69	90	20

Supplementary Table 5. Cross between bisexual Td-HR strain males and thelytokous TdT-HR strain females to determine the possibility of suppressor gene.

Line of F0	F1 (daughter)		F2 (granddaughter)	F3
Td-HR♂ × TdT-HR♀	♀	♂		
1	51	0	all ♀	all ♀
2	60	0	all ♀	all ♀
3	57	0	all ♀	all ♀
**4**	**5**	**37**	all ♀	all ♀
5	46	0	all ♀	all ♀
6	52	0	all ♀	all ♀
7	0	0	NA	NA
8	64	0	all ♀	all ♀
9	72	0	all ♀	all ♀
10	70	0	all ♀	all ♀
11	88	0	all ♀	all ♀
12	56	0	all ♀	all ♀
**13**	**13**	**36**	all ♀	all ♀
14	67	0	all ♀	all ♀
15	55	0	all ♀	all ♀
16	67	0	all ♀	all ♀

17	0	0	NA	NA
18	69	0	all ♀	all ♀
**19**	**10**	**48**	all ♀	all ♀
20	50	0	all ♀	all ♀
21	57	0	all ♀	all ♀
22	40	0	all ♀	all ♀
23	44	0	all ♀	all ♀
**24**	**0**	**18**	NA	NA
25	75	0	all ♀	all ♀
26	62	0	all ♀	all ♀
27	53	0	all ♀	all ♀
28	54	0	all ♀	all ♀
29	59	0	all ♀	all ♀
30	0	0	NA	NA
31	33	0	all ♀	all ♀
32	56	0	all ♀	all ♀
33	65	0	all ♀	all ♀
34	0	0	NA	NA
35	37	0	all ♀	all ♀
36	53	0	all ♀	all ♀
37	48	0	all ♀	all ♀
38	65	0	all ♀	all ♀
39	54	0	all ♀	all ♀
40	0	0	NA	NA
41	61	0	all ♀	all ♀
42	32	0	all ♀	all ♀
**43**	**1**	**48**	0	NA
**44**	**7**	**43**	all ♀	all ♀
45	70	0	all ♀	all ♀
46	66	0	all ♀	all ♀
**47**	**16**	**26**	all ♀	all ♀
48	55	0	all ♀	all ♀
49	49	0	all ♀	all ♀
50	68	0	all ♀	all ♀
51	0	0	NA	NA
**52**	**16**	**38**	all ♀	all ♀
53	57	0	all ♀	all ♀
54	60	0	all ♀	all ♀
55	38	0	all ♀	all ♀
56	71	0	all ♀	all ♀
**57**	**11**	**45**	all ♀	all ♀
58	49	0	all ♀	all ♀
59	21	0	all ♀	all ♀
60	62	0	all ♀	all ♀

Note: Designations in bold represent the lines with high male proportion.

Supplementary Table 6. Cross between males from high male biased lines in Supplementary Table 5 and females from thelytokous TdT-HR strain.

Hybrid lines		F2		F3		F4	
♂ line (from Table 5)	TdT-HR♀	♀	♂	♀	♂	♀	♂
4	1	2	18	11	14	10	42
	2	9	21	0	0	NA	NA
	3	10	57	8	50	4	25
	4	0	56	10	33	20	76
	5	25	38	0	62	22	39
13	6	3	50	15	53	0	38
	7	1	6	11	31	28	34
	8	9	41	20	26	5	37
	9	0	0	NA	NA	NA	NA
	10	18	64	3	38	4	66
19	11	10	25	1	47	0	0
	12	8	40	17	18	7	35
	13	5	38	8	55	0	51
	14	2	52	6	55	11	32
	15	28	47	13	68	19	40
24	16	0	0	NA	NA	NA	NA
	17	11	29	9	54	20	37
	18	0	35	9	60	19	37
	19	9	36	18	52	16	33
	20	4	51	0	46	14	41
43	21	21	30	25	36	8	15
	22	12	56	5	59	23	30
	23	1	46	14	34	0	0
	24	0	65	4	44	16	34
	25	6	39	28	34	18	48
44	26	0	53	5	56	8	66
	27	0	0	NA	NA	NA	NA
	28	13	45	0	58	0	9
	29	7	47	10	61	19	14
	30	22	36	18	39	10	42
47	31	33	34	1	52	0	0
	32	0	47	21	23	0	6
	33	3	33	9	38	20	22
	34	7	27	9	26	0	68
	35	0	48	0	0	NA	NA

52	36	20	41	0	0	NA	NA
	37	1	57	8	53	0	0
	38	0	0	NA	NA	NA	NA
	39	17	32	11	55	13	54
	40	1	64	7	28	0	62
57	41	10	68	16	22	10	30
	42	0	44	0	59	2	41
	43	14	42	30	20	2	49
	44	0	0	NA	NA	NA	NA
	45	19	27	8	39	12	48

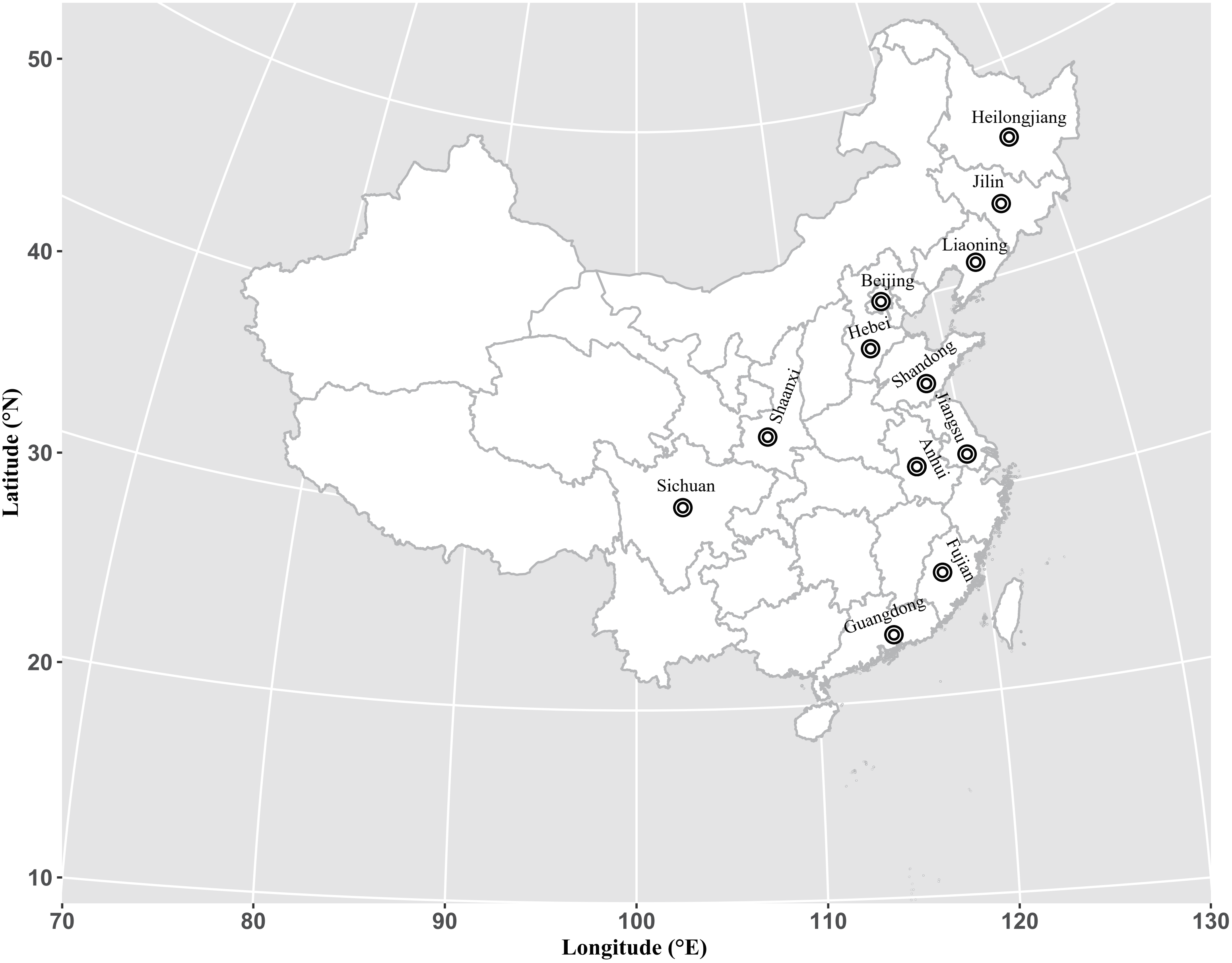

Supplementary Figure 1. Spatial distribution of *Trichogramma dendrolimi* populations in China.

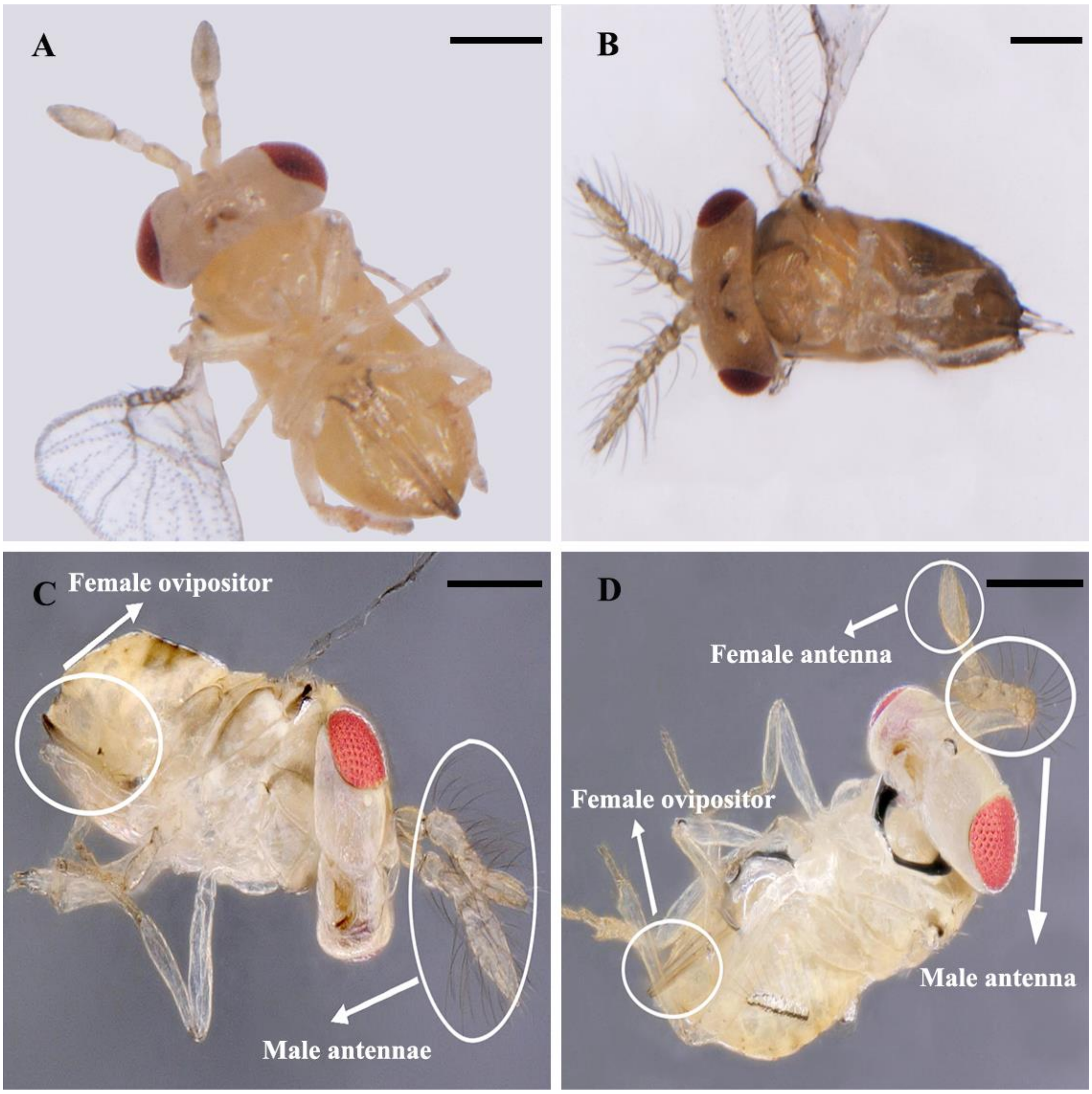

Supplementary Figure 2. Female (A), male (B) and two types of intersex (C and D) *Trichogramma dendrolimi*. The black bars equal 100 μm.

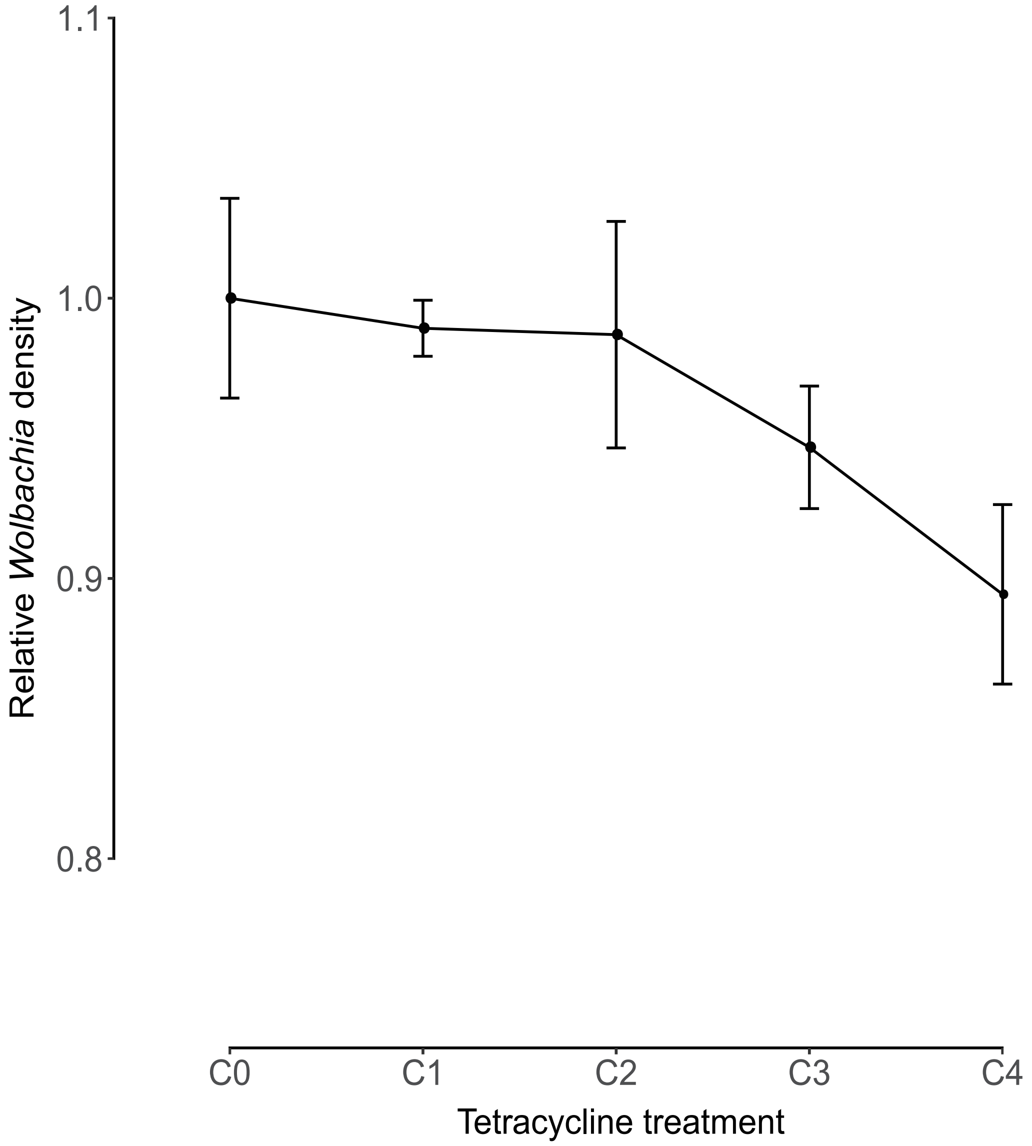

Supplementary Figure 3. *Wolbachia* titer under different concentrations of tetracycline. C0 is the no tetracycline treatment control, C1 to C4 represent 0.0001, 0.001, 0.01 and 0.1 mg/ml tetracycline respectively. The single copy gene *wsp* and *forkhead* (of host) were used to estimate the relative titer of *Wolbachia*[1,2]. We performed quantitative PCR (qPCR) in a Bio-Rad CFX96 Real-time PCR Detection System (Bio-Rad, Hercules, California, USA) with new primers designed using Primer 5.0 software (Primer-E LTd., Plymouth, UK) according to the sequences

obtained in current study (*wsp*) and from GenBank (*forkhead*, KX066243). The specificity and efficiency of primers were checked prior to estimating *Wolbachia* titer. Condition for both *wsp* (Forward: 5'-CAACAAGTAAAGAGGAGGAT-3'; Reverse:5'-GTAAAGCCCTTCAACATC-3') and *forkhead* primers (Forward: 5'-CTACGCCGATCTCATAACGC-3'; Reverse: 5'-TGCTGTCGCCCTTGTCCT-3') was: 95 ℃ for 5 min, then 40 cycles of 95 ℃ for 15 s and 55 ℃ for 45 s. Each qPCR was performed in a total volume of 20 μL containing 10 μL 2 × SYBR Green Supermix (Bio-Rad, Hercules, California, USA), 0.5 μL each of forward and reverse primers (10μM), 1 μL DNA template and 8 μL ddH_2O. Genomic DNA was extracted as described in main article. A third technical replicates was completed for each sample. The relative quantity of *Wolbachia* was calculated by $2^{-\Delta\Delta}$CT method[3]. Then a GLM was used to compare the *Wolbachia* titer among different tetracycline treatments based on a Gaussian distribution. The result showed that higher tetracycline concentrations resulted in lower *Wolbachia* titer (GLM, $\chi^2 = 37.117$, d.f. = 1, $P < 0.0001$).

1 Braig HR, Zhou W, Dobson SL, O'Neill SL, Cloning and characterization of a gene encoding the major surface protein of the bacterial endosymbiont *Wolbachia pipientis*. *J Bacteriol* **180**:2373-2378 (1998).

2 Tulgetske GM, Investigations into the mechanisms of *Wolbachia* induced parthenogenesis and sex determination in the parasitoid wasp, *Trichogramma*. PhD Dissertation, UC Riverside, Riverside, California (2010).

3 Livak KJ, Schmittgen TD, Analysis of relative gene expression data using real-time quantitative PCR and the $2^{-\Delta\Delta CT}$ method. *Methods* **25**:402-408 (2001).
